# Near-Complete Genome Sequence of an Enterovirus Species F Isolate Recovered from Sewage in Nigeria

**DOI:** 10.1128/MRA.00094-20

**Published:** 2020-05-28

**Authors:** T. O. C. Faleye, M. I. Ifeorah, O. A. Olayinka, B. Oluremi, U. E. George, O. A. Arowolo, E. C. Omoruyi, E. Donbraye, A. O. Oyero, O. M. Adewumi, J. A. Adeniji

**Affiliations:** a Center for Human Virology and Genomics, Department of Microbiology, Nigerian Institute of Medical Research, Lagos, Nigeria; b Department of Virology, Faculty of Basic Medical Sciences, College of Medicine, University of Ibadan, Ibadan, Nigeria; c Department of Medical Laboratory Sciences, Faculty of Health Sciences and Technology, University of Nigeria, Nsukka, Nigeria; d Department of Pharmaceutical Microbiology, Faculty of Pharmacy, University of Ibadan, Ibadan, Nigeria; e Department of Biological Sciences, Redeemer’s University, Ede, Nigeria; f Viral Vaccines Production Division, National Veterinary Research Institute, Vom, Plateau State, Nigeria; g Institute of Child Health, College of Medicine, University of Ibadan, Ibadan, Nigeria; h Department of Medical Microbiology and Parasitology, Obafemi Awolowo University, Ile-Ife, Nigeria; i WHO National Polio Laboratory, University of Ibadan, Ibadan, Nigeria; j Infectious Disease Institute, College of Medicine, University of Ibadan, Ibadan, Nigeria; Portland State University

## Abstract

Here, we describe the near-complete genome of an enterovirus F (EV-F) isolate from Nigeria. The obtained sequence was 7,378 nucleotides (nt) long and encodes 2 open reading frames (ORFs), an upstream ORF (uORF; 56 amino acids [aa]) and a polyprotein ORF (ppORF; 2,167 aa). Both ORFs overlap but are in different reading frames, with the uORF in a +1 reading frame relative to the ppORF.

## ANNOUNCEMENT

Enteroviruses (EVs) are members of the genus *Enterovirus*, family *Picornaviridae*, order *Picornavirales*. In 2018, we described the genome of the first EV-E isolate from Nigeria, which was recovered from a sewage sample collected in 2017 in Borno State, Nigeria (1). Here, we describe the genome of the first EV-F isolate from Nigeria.

The isolate was recovered from a sewage sample collected in Enugu State, Nigeria, in 2018 as part of the environmental surveillance program for poliovirus in the country. Specifically, 500 ml of a sewage sample collected by the grab method was concentrated using the two-phase separation protocol and inoculated into both the L20B and rhabdomyosarcoma (RD) cell lines (2). The isolate replicated in both cell lines, producing characteristic EV cytopathology, and was consequently subjected to intratypic differentiation real-time reverse transcription-PCR (rRT-PCR) assays using the ITD 5.0 rRT-PCR kit (3). The isolate was negative for all the assays and was therefore classified as not poliovirus and not an enterovirus (NEV). Thereafter, RNA was extracted from the isolate and cDNA was made using a total RNA extraction kit and SCRIPT cDNA synthesis kit (Jena Bioscience, Germany), respectively. The cDNA was then shipped to a commercial facility (MR DNA, TX, USA), where library preparation and Illumina sequencing were performed. Library preparation was conducted using the TruSeq RNA LT sample preparation kit (Illumina), sequencing was performed in a paired-end (2 × 150-bp) format using the HiSeq system (Illumina), trimming was done using Trimmomatic v1.2.14, and Kiki v0.0.9 was used for *de novo* assembly as previously described (1). All contigs were subjected to a BLASTn search and thereby identified and annotated. All software was used with default settings.

A contig similar to EV genomes (7,378 nucleotides [nt] long) was recovered from 0.14% (4,295 reads) of the 2.97 million reads generated (coverage, 75.62×). The contig has a G+C content of 51.05%. A BLASTn search of the contig against the NCBI database showed it to be most similar (79.44% identity) to GenBank accession number KC748420 (an EV-F strain recovered from an alpaca in the United States in 2007). The contig has both expected open reading frames (ORFs). The upstream ORF (uORF) (4) has 56 amino acids (aa) and overlaps a polyprotein ORF (ppORF; 2,167 aa). However, both are in different reading frames, with the uORF in a +1 reading frame relative to the ppORF. The ppORF is 94.83% similar to an EV-F strain (QDQ46321.1) detected in goats in China in 2018 (5).

To the best of our knowledge, this is the first EV-F genome sequence described in Nigeria and possibly in sub-Saharan Africa. We hope that this genome sequence serves as a reference for better characterization of EV-F diversity in the region. Furthermore, considering that this EV-F member replicates efficiently in both human (RD) and mouse (L20B) cell lines, it is important that its receptor usage in these cell lines be delineated and compared with that in bovine cell lines (e.g., MDBK), on which most EV-E and EV-F were traditionally isolated (6). Understanding this might help illuminate the mechanism of species jump in zoonotic enteroviruses.

### Data availability.

The genome described here has been deposited in the SRA under BioProject accession number PRJNA514299 and GenBank accession number MN650196.
